# Severe Acute Respiratory Syndrome Coronavirus 2 (SARS-CoV-2) Infection: Triggering a Lethal Fight to Keep Control of the Ten-Eleven Translocase (TET)-Associated DNA Demethylation?

**DOI:** 10.3390/pathogens9121006

**Published:** 2020-11-30

**Authors:** Sofia Kouidou, Andigoni Malousi, Alexandra-Zoi Andreou

**Affiliations:** 1 Lab of Biological Chemistry, Medical School, Aristotle University of Thessaloniki, 541 24 Thessaloniki, Greece; andigoni@auth.gr; 2 Institute of Physical Chemistry, University of Münster, 481 49 Münster, Germany; alexandra.andreou@uni-muenster.de

**Keywords:** COVID-19, SARS-CoV-2, DNA methylation, TET enzymes

## Abstract

The extended and diverse interference of severe acute respiratory syndrome coronavirus 2 (SARS-CoV-2) in multiple host functions and the diverse associated symptoms implicate its involvement in fundamental cellular regulatory processes. The activity of ten-eleven translocase 2 (TET2) responsible for selective DNA demethylation, has been recently identified as a regulator of endogenous virus inactivation and viral invasion, possibly by proteasomal deregulation of the TET2/TET3 activities. In a recent report, we presented a detailed list of factors that can be affected by TET activity, including recognition of zinc finger protein binding sites and bimodal promoters, by enhancing the flexibility of adjacent sequences. In this review, we summarize the TET-associated processes and factors that could account for SARS-CoV-2 diverse symptoms. Moreover, we provide a correlation for the observed virus-induced symptoms that have been previously associated with TET activities by in vitro and in vitro studies. These include early hypoxia, neuronal regulation, smell and taste development, liver, intestinal, and cardiomyocyte differentiation. Finally, we propose that the high mortality of SARS-CoV-2 among adult patients, the different clinical symptoms of adults compared to children, the higher risk of patients with metabolic deregulation, and the low mortality rates among women can all be accounted for by the complex balance of the three enzymes with TET activity, which is developmentally regulated. This activity is age-dependent, related to telomere homeostasis and integrity, and associated with X chromosome inactivation via (de)regulation of the responsible *XIST* gene expression.

## 1. Introduction

Our knowledge regarding the epidemiological data of coronavirus disease (COVID-19) is rapidly increasing [1,2]. Proteomic analysis of severe acute respiratory syndrome coronavirus 2 (SARS-CoV-2) and its homology to severe acute respiratory syndrome coronavirus (SARS-CoV) [3,4] revealed the critical role of the Spike (S) protein with an ACE2 binding region [5]. ACE2 is regarded as responsible for SARS-CoV and other viruses’ entry into the lungs [6]. This fact has led to extensive work on the design of related drugs. However, efforts for targeting the COVID-19 pandemic require comprehension of the viral mechanisms and its targets, including those associated with its “unique” or “unusual” and possibly less troubling, accompanying symptoms. This is particularly important, since there is an extensive ongoing discussion with regards to the long-lasting viral consequences and the threat that the present pandemic will be followed by an equally serious forthcoming one: The association with an increase of Alzheimer’s and neurodegenerative diseases related to the SARS-CoV-2 damage of the nervous system [7]. Conditions involving metabolic deregulations [8], as well as obesity, are considered COVID-19 risk factors.

In the following sections, we present evidence, associating the frequent, but “unique” symptoms of SARS-CoV-2 infection with the mechanism of DNA demethylation by ten-eleven translocase (TET) activity. This activity is responsible for differentiation and tissue-specific regulation of gene expression, regulation of X chromosome inactivation, age-dependent and telomere-related activity, and finally, regulated in vivo by a process similar to that disrupted by SARS-CoV in the process of ACE2 deactivation.

## 2. Evidence for TET Deregulation Associated with Viruses and Hypoxia

Evidence of viral RNA interference in TET2 regulation has been previously reported in the case of Human T-lymphotropic virus type 1 [9] and Epstein-Barr Viral infection [10].

A common mechanism of viral interference involving TET activity is associated with the TET2-dependent IFITM3 deregulation [11]. IFITM3 plays a protective role and reduces the susceptibility of the host membrane to the viruses. In the case of HIV infection, this mechanism involves TET activity deregulation mediated by proteasome activity. Hypoxia inhibitory factor (HIF) activity, which is deregulated by von-Hippel Lindau tumor suppressor (pVHL), is also mediated by TET deregulation through proteasomal degradation [12]. The above mechanism probably shares common grounds with the mechanism described by Guallar et al. [13], which accounts for the role of TET2 in the viral regulation of endogenous viral RNAs. According to the authors, TET2 that lacks a DNA recognition domain, binds to viral RNA specifically, depending on the RNA structural characteristics. The RNA interaction with TET is necessary for exerting the TET activity. Guallar et al. showed that in the case of endogenous viral RNAs, the host paraspeckle component 1 (PSPC1) mediates the interaction of viral RNAs with TET2, leading to viral inactivation [13]. Finally, hypoxia is also proposed as a leading factor associated with TET1 and TET3 silencing. Actually, hypoxia targeted genes regulate, together with the cellular metabolites succinate and fumarate [14], the TET-associated epigenetic response via a proteasome-mediated mechanism.

## 3. TET Activity, Biological Significance, and Characteristics of Target Sequences

Extensive studies during the last years have documented the role of DNA cytosine methylation in cell differentiation, splicing, and alternative splicing [15,16,17]. DNA cytosine methylation is a non-reversible reaction for which DNA methyltransferase 1 (DNMT1) and DNA methyltransferase 3 (DNMT3) are responsible [18].

The activity of TETs is mostly responsible for DNA demethylation [19]. TETs 1–3 are members of the 2-oxoglutarate-dependent dioxygenase (2-OGDO) family. TETs require the presence of 2-oxoglutarate and O_2_ as obligatory substrates and the presence of Fe^2+^ as a cofactor. 2-OGDO enzymes are sensors of the energy metabolism, since 2-oxoglutarate is an intermediate activator of the Krebs cycle, whereas succinate and fumarate are potent inhibitors of 2-OGDO enzymes [20]. O_2_ availability and iron redox homeostasis control the activities of 2-OGDO enzymes in tissues and are strongly influenced during the infection [21,22]. The product of TETs is 5-hydroxymethyl cytosine (5hmC) that is processed by formylation and carboxylation [19]. 5-methyl cytosine (5mC) oxidation products regulate several life-associated mechanisms [23,24], including developmental processes and aging [24], neurological disorders and DNA repair [25], as well as cancer [23,26]. 5hmC has been mainly traced in human tumors, but it is also frequently associated with brain function, such as in brain repair mechanisms taking place after a stroke [27]. TET activity is particularly important in embryonic stem cells [28]. TET3 mutations are associated with lethality [29], and TET1 mutations are lethal in all but in-bred mice [29,30]. Both TET1 and TET3 are expressed in mice hypothalamus, but their activity is attenuated by age and activated by exercise [31]. In epithelial cells, TET1 is involved in activation of the hypoxia-induced, epithelial-mesenchymal transition [32].

Hydroxymethylated cytosines are mostly associated with transcription factor binding sites [33]. In a recent study on the characteristics of the 5hmC-containing sequences in normal liver, we showed [34] that TET activity is most frequently exerted in sequences which contain the TGGGA and TCCCA pentanucleotides arranged so that they could form hairpins under certain conditions and next to G-quadruplexes. These formations can be recognized by bivalent zinc finger proteins introducing DNA distortion. The role of TET activity and DNA hydroxymethylation in proximity with these motifs is probably to relax such a distortion. This mechanism is lost in cancer.

### 3.1. Zinc Fingers Involved in HIV- and SARS-CoV-Related Activity and Proteolytic Degradation of Host Proteins

Zinc finger proteins are commonly employed by viruses, such as HIV and SARS-CoV, for manipulating the human genetic material. One such example is the 3ECTO zinc finger protein of SARS-CoV. Similar zinc fingers are common in coronaviruses, such as Middle Eastern Respiratory Syndrome (MERS-CoV), but also in other viruses, such as HIV1 [35]. 3ECTO is located in Nsp3 of SARS-CoV, a non-structural protein that also possesses papain protease activity. The functional role of 3ECTO with its papain-like protein domain (PLPRO) is not well understood. However, this zinc finger and the related PL2pro (papain protease activity) are essential for SARS-CoV activity and responsible for reducing the activity of vital host proteins [36]. PL2pro, similarly to SARS-CoV and other coronaviruses, participates in viral entry into the host via the ACE2 receptor [5].

All three TET analogs are monoubiquitinylated by CRL4 (VprBP), an E3 ubiquitin ligase with multiple components (a DNA binding component, DDB1; a ubiquitin ligase 4A/B, CUL4; DCAF1, a substrate recognition component of E3 ubiquitin-protein ligase and atypical serine/threonine-protein kinase; and finally, RBX1, a ring-binding component [37]). Under physiological conditions, ubiquitylation occurs at a highly conserved lysine residue, which plays a regulatory role in TET activity and promotes its binding to DNA [38]. TMPRSS2, a protease with an ill-defined biological role [39], is involved in the regulation of CRL4 (VprBP) via its RBX1 component. Biological evidence for the RBX1 and TMPRSS2 association and its role in the activity of the above ligases (Cullin-RING E3 ligases) results from the TMPRSS2-ERG translocation [30,31]. This evidence is in support of the TMPRSS2 involvement in TET 1-3 activity or expression balance deregulation.

A characteristic system of viral intervention and host surveillance deregulation has been described for the destruction of SARS-CoV. The virus is thought to interfere with the cellular E3 ubiquitin ligase ring-finger system by using a two-component system: The SARS-unique domain (SUD), which can bind to a G-quadruplex through a CHY protein domain, and the papain-like protease (PL(pro)). This interaction interferes with ubiquitin-dependent p53 antiviral cellular activity, and coronavirus escapes replication surveillance [40]. P53 activity interference by SARS infection is mediated by p21 down-regulation [40]. Viral protease activity is also responsible for protein inactivation by processes related to ubiquitin- or ubiquitin-like gene, known as interferon-stimulated gene 15 (ISG15) [41,42]. Furthermore, TET2 acetylation is observed during oxidative stress, together with simultaneous and reversible methylation and 5hmC increase [43]. Provided that one characteristic of TETs’ targets are G-quadruplex sequences [34], it could be expected that SARS-CoV-2, of similar structure, might also contribute to the proteolytic deregulation of TETs.

The association of the ACE2 and TET deregulation mechanisms by SARS-CoV-2 has been very recently verified by Chen et al. [44]. These authors reported that the E3 ubiquitin ligase system is deregulated by SARS-CoV-2. Actually, Chen et al. proposed that endogenous TMPRSS2 regulation is subject to the E3 ubiquitin ligase components DDB1 and CUL4-associated factor 1 (DCAF1), both involved in TET degradation [44]. It has been shown that IFITM3 is a target of TMPRSS2 [45].

The biological roles of TET1 paralogs, TET2 and TET3, have not been sufficiently elucidated to date, and there are no references with respect to the association of TET activity with factors involved in the SARS-CoV-2 activity. TET1 mutations are lethal in normal mice (non-inbred) and act through repressing the majority of epiblast target genes independently of methylation changes [30]. In vivo deactivation of TET2 has been attributed to proteolytic activity that depends on the binding of another zinc finger protein, IDAX (also known as CXXC4), to DNA [46]. The IDAX zinc finger binds unmethylated DNA and interacts directly with the catalytic domain of TET2. This process is followed by TET2 protein degradation, a yet ill-defined mechanism that is related to the IDAX and TET2 interaction through their zinc finger domains and with the unmethylated DNA. Down-regulation of TET is dependent on IDAX expression and activity, as well as on zinc finger interactions.

All the above pathways have been investigated as targets of antiviral drugs. Halofuginone, a drug that targets the ubiquitin-related ligase system involved in the degradation of the above endogenous proteins (TETs and TMPSS2), was shown to be an active SARS-CoV-2 inhibitor [44]. Another example of a compound that targets zinc fingers, such as the RNA-binding nucleocapsid protein p7 (NCp7) of HIV1, is bananin, which has been shown to provide protection against SARS-CoV [47].

### 3.2. Aging, DNA Methylation, and TET Activity

Adjustment of DNA methylation to developmental and environmental “stress” conditions becomes inefficient with age. Biological clocks, such as that proposed by Horvath [48], provide a weighted average of methylation deviations in critical loci of an individual’s genome as indicators of human age. An analytical study of the characteristics of the DNA sequences included in this biological “clock” reveals that critical and representative methylation changes are observed in DNA sequences that have the potential to “fold” under certain conditions [49]. One category of structural elements that is important for DNA folding is guanosine-rich sequences with specific guanosine distribution patterns known as G-quadruplexes [50]. Another category of structural elements is the palindromes that can, similarly to single nucleotide polymorphisms (SNPs), influence genome structure and expression depending on environmental factors, such as diet [51]. Sequences with the potential to “fold” and create these DNA micro-structures are also frequent in regions of epigenetic control by the TET demethylating activity and contribute to methylation “adjustments” [34]. The 5hmC presence is cell-selective and perturbed in pathological conditions, such as cancer [52].

TET distribution, dynamics, and activity in the brain has been extensively investigated [53]. During synaptogenesis, 5hmC accumulates in the euchromatin, and colocalizes with PolII that mediates RNA transcription. Due to this process, TET activity mediates the function and development in the brain. The presence of TET is also critical for the regulation of bivalent promoter methylation in stem cell differentiation [54]. TET2, and possibly the other TET enzymes, are also inactivated by aging, due to mutations accumulating in otherwise healthy individuals [55]. Actually, cytosine hydroxymethylation is probably more affected by aging, since it is dependent on the abundance of its 5mC substrate and affected by TET activity modifications, due to fluctuations of the expression levels of the different TET family members.

G-quadruplexes are also involved in 5hmC regulation. The distribution of G-quadruplexes among human chromosomes is uneven. Stable G-quadruplex structures are found in sub-telomeres, gene bodies, and gene regulatory regions [56]. G-quadruplexes stabilize the binding of ligands that can modulate transcription in several target genes [56]. The X chromosome is “poor” in G-quadruplexes (unpublished data) and in hydroxymethylated regions [34], including regions involved in metabolic processes (coding for mitochondrial and ribosomal proteins). An exception is the *XIST* gene, located in the chromosome X; one of *XIST*’s products, is a long non-coding RNA that is responsible for X chromosome inactivation, while its shorter product, XPi2, stimulates the expression of genes by attenuating G-quadruplex formation [57].

Telomeres are considered the “guardians” of the genome. Telomere attrition is strongly involved in cell renewal, genome stability, and DNA repair, while telomere shortening is considered a characteristic of aging to which both genetic and epigenetic factors play an active role [58,59]. TETs also play a critical role in telomeric stability [60].

### 3.3. Common and Unique Symptoms Associated with SARS-CoV-2 Infection

Several recent reports refer to various unusual symptoms associated with the SARS-CoV-2 infection and frequently observed among patients. In addition to the respiratory distress, which is very common among individuals affected by SARS-CoV-2 and cardiac injury, other symptoms, such as various neurological symptoms [61,62], viral-associated olfactory loss [63], and ataxia [61] are also very frequently reported. Mao et al. [61] also reported that admitted patients with Acute Respiratory Distress Syndrome (ARDS), due to COVID-19, frequently showed symptoms of encephalopathy, prominent agitation, confusion, and corticospinal tract signs (69%), when the neuromuscular blockade was discontinued, in addition to cardiovascular complications and very common early hypoxia [64]. Liver injuries were also common, while death was frequently related to immune-mediated cytokine release syndrome [21]. Additional epidemiological characteristics of the disease are also unusual: a very significant, but less frequently discussed, difference in mortality between males and females [65]; frequent disease-associated manifestations among middle-aged and elderly individuals [66] compared to children and adolescents; good anticipated outcome among children [67]. As a result, infection rates are still unclear in younger ages, and guidelines concerning the treatment of infected children are still unclear [68]. One more “puzzling” finding is that the SARS-CoV-2 epidemic is reportedly associated with a high incidence of a severe form of Kawasaki disease-like symptoms among children [69]. Finally, skepticism is frequently expressed with regards to the medium- and long-term neurological consequences among survivors following long-lasting treatment for SARS-CoV-2 infection, and the possibility that such an outcome might lead to an equally, or more serious, delayed neurological pandemic with major public health impact [7].

### 3.4. TET Activity, Common SARS-CoV-2 Symptoms, and Epidemiological Characteristics

The association between TET2 and pulmonary function has been previously studied by Potus et al. [70]. The authors identified TET2 mutations and expression in mononuclear cells and showed the complex role, as well as the variety of symptoms, in a cohort study of patients with Pulmonary Arterial Hypertension (PAH) in the absence of mutations in other PAH-related genes. TET2-knockout mice also developed PAH, and inflammation related to cytokine elevation, including IL-1β [70].

However, according to previous studies, the activity of the three TET enzymes is diverse or even contradictory. For example, the role of TET1 and TET2 osteogenesis and adipogenesis can be opposite [71], e.g., TET1 was found to repress both osteogenesis and adipogenesis, while TET2 was found to promote the opposite processes. Although it is possible that only one of the TET enzymes might be affected by SARS-CoV-2 and might be responsible for their tissue specificity and its association with different developmental states, complex mechanisms involving the expression of different TETs are also conceivable. Thus, the age-related response among children, adolescents, and older adults to the viral infection could be associated with differential expression of TETs at different ages and developmental stages.

#### 3.4.1. Neurological Symptoms

Epigenetic modifications have a very significant impact on neuronal function. Evidence that TET activity and 5hmC presence are associated with neuronal function in humans results from neuronal degeneration studies [72]. Ataxia is frequent among COVID-19 patients and is associated with DNA hydroxymethylation in mouse models with fragile X-associated tremor/ataxia syndrome [73]. Global changes in 5hmC modification characterize traumatic spinal cord tissues [74]. On the contrary, restoration of spinal cord injury-induced in rats [75] following ascorbic acid supplementation was associated with 5hmC level increase and mRNA level increase of all TET gene family members.

Loss of taste and smell is another frequent symptom among patients that are probably associated with deregulation of epigenetic modifications in the olfactory bulb located in the brain [76]. The olfactory bulb is responsible for the development of smell and taste in association with 5mC and 5hmC modifications. Actually, it has been proposed that site-specific development of non-CpG cytosine hydroxymethylation is developed during this process, in addition to hydroxymethylation in CpG sites. It has also been proposed that non-CpG hydroxymethylation might be related to active cytosine modification turnover and fine-tuning of gene expression in the mammalian brain [76]. In a model system of honey bees, olfactory training has also been associated with the presence of 5hmC [77]. The topology of epigenetic modifications, and the presence of gene bodies which were enriched in CpGs with variable epigenetic modifications (5mC/5hmC), provided evidence for the impact of TET modifications in neuronal cell fine-tuning [77].

#### 3.4.2. Liver and Intestinal Dysfunctions Associated with SARS-CoV-2 Infection and Adverse Pulmonary Vascular Remodeling

The liver is a frequent “target” of SARS-CoV-2 [78], and intestinal dysfunction is also common among patients. TET activity appears to be critical for liver function, activation, and differentiation [79]. Studies in non-alcoholic fatty liver disease reveal that TET probably regulates liver biogenesis by controlling mitochondrial biogenesis [80]. Moreover, it has been proposed that the common 5hmC-related gene expression profiles in adult mouse brain and liver are coordinated by common TET activity [81].

TET activity in the form of promoter hydroxymethylation and TET1 expression also regulates expression in intestinal stem cells (ISC), through the cancer-associated Wnt signaling pathway [82]. Intestinal development in TET1-deficient mice is associated with growth-retardation, partial postnatal lethality, and reduced organoid-forming capacity [82]. Thus, in addition to the negative impact of TET2 decreased expression and its association with PAH [70], TET activity can negatively influence the function of several organs and systems in which functional irregularities are observed among COVID-19 patients.

#### 3.4.3. TET Activity and Cardiac Dysfunction: Associations with the Krebs Cycle Oxidation, Hypoxia, and Oxidative Stress

TET is a major regulator of gene expression activity in cardiomyocytes [83]. It has been proposed that in cardiomyocytes, TET acts as a modulator of transcript length and abundance, as well as gene-body epigenetic modifications. This process is correlated with the presence of the TET product, i.e., 5hmC. TET, similarly to other 2-oxoglutarate-dependent dioxygenases, is a sensor of the energy metabolism [20], associated with the activation of the citric acid cycle. Thus, TET mediates metabolically regulated DNA epigenetic processes [84] in the cardiomyocyte, regulating differentiation, splicing, and alternative splicing in these cells [85]. Oxidative stress is associated with the reduction of TET activity and increased global levels of H3K4me3 and H3K27me3 [86]. Succinate and fumarate are regulators of TET activity [14] and are allosteric regulators of the alpha-ketoglutarate-dependent dioxygenase family enzymes [87].

The association of hypoxia with TET activity has been shown in tumors, where TET activity is repressed, due to hypermethylation [88] and regulated by two intermediates of the Krebs cycle: fumarate and succinate. Reduction of the TET activity and hypoxia in cancer, is considered responsible for up to half of the hypermethylation events. TET silencing has different effects on the hypoxia-inducible genes [14]. 2-oxoglutarate-dependent dioxygeneases, in addition to oxygen modulation [89], are generally considered as regulators of neuronal death. Although silent hypoxia is not a very frequent symptom associated with the SARS-CoV-2 infection [90], hypoxia is very common among SARS-CoV-2 patients [22] and closely related to TET1 and TET2 activities [91].

#### 3.4.4. Aging, Telomeres, TETs and DNA Methylation

Provided that TET regulates the activation of bivalent (or bimodal) promoters in embryonic stem cells and is involved in development [54], it could be expected that TET activity could be associated with aging. TET activity is indeed a principle epigenetic factor responsible for telomere maintenance and structure [92]. Although the roles of different TETs are redundant, it has been shown that TET activity is associated with telomeric elongation [58], and TET1 is particularly involved in telomeric regulation in the post-implantation mouse embryo [30]. According to our recent findings [34], TET activity probably contributes to the helical flexibility required to form different conformations during the binding of bivalent transcription factors. The helical flexibility introduced by cytosine hydroxymethylation also contributes to the “coiling” of the telomeric TTAGGG repeat [30], which can assume alternative 3D telomeric structures [93]. The compromised TET function, associated with the SARS-CoV-2 presence, would be expected to enhance telomere attrition that is observed among elderly patients in intensive care units [94]. Indeed, the morbidity associated with SARS-CoV-2 has been attributed to telomeric compromise in lymphocytes [95].

Another mechanism associated with aging and affecting the telomere length, involves the process of inflammation [87] and the deregulation of inflammatory cytokine expression (e.g., IFN-γ, IL-6, and TNF-α). It is evident that TET activity could play a regulatory role in this process, since it is involved in telomeric integrity and in the regulation of cytokine expression by modifying these transcription factor binding sites [34].

In view of the above, it can be concluded that telomeric changes are subject to TET-associated epigenetic regulation, and thus, might also be responsible for the high death rates observed among the elderly SARS-CoV-2 patients. Epigenetic modification in telomeres is also regulated by cytosine methylation that is universally compromised with age. Its weighted presence is reflected by the presence of 5mC in selected CpGs. It is, however, unclear to which extent the 5hmC decrease reported for the elderly in peripheral blood T cells [96], is a consequence of variations in DNA methylation.

Finally, TET2 expression is subject to developmental and hormonal regulation in some organs. Kurian et al. [97] reported that TET2 activity increases in the developing mouse preoptic area-hypothalamus and is substantially higher in mature, compared to immature animals. Changes in TET2 expression levels resulted in modified TET2 genome binding and in histone 3 lysine 4 trimethylation (H3K4me3) abundance changes at the gonadotropin-releasing hormone (GnRH) promoter. Symptoms observed in mice with selective disruption of TET2 in GnRH-releasing neurons, indicated that the role of TET2 is very complex, probably associated with mitochondrial function, and RNA metabolism and involved in the maintenance of GnRH neuronal function in adults.

#### 3.4.5. Epigenetic Modifications in the X Chromosome, Its Inactivation, and Sex-Dependent TET Regulated Activities. The Cytosine Storm

The X chromosome, in which more than 75% of the genes coding for mitochondrial and ribosomal proteins reside, exhibits 5hmC levels that are considerably lower compared to those of autosomes [81]. This characteristic of the X chromosome might be related to its unusual structure and its very low G-quadruplex content, and lower expected nucleosome occupancy [34]. However, the expression of the *XIST* gene (located in the X chromosome) that is responsible for the X chromosome deactivation, is regulated by a 5hmC-dependent mechanism [98]. The *XIST* gene codes for a long non-coding RNA (lncRNA), which is indispensable for the X chromosome inactivation [99].

The cytokine storm, which is very frequently responsible for SARS-CoV-2 associated deaths among male patients [100,101], might also be related to loss of PTX3 transcription factor regulation. *PTX3* is the target of hydroxymethylation by TET and plays a role in the regulation of innate resistance to pathogens, inflammatory reactions, as well as clearance of self-components and female fertility (PTX3_HUMAN,P26022 UniProtKB/Swiss-Prot; in [34]). All previously analyzed factors could account for the high mortality caused by SARS-CoV-2. However, the relatively lower mortality rates recorded for women could be associated with the loss of *XIST*-related X chromosome deactivation, which would confer increased protection towards TET activity deregulation among females and regulation of promoters responsible for cytokine control [34].

Proteins associated with metabolic processes and under the control of Jumonji C (JmjC) domain are also TET-regulated and play an important epigenetic role [30,81]. Some of the SARS-CoV-2 symptoms have also been reported for young Kawasaki patients, in which a set of CpGs exhibits discretely different DNA methylation levels [102]. However, many other SARS-CoV-2 associated symptoms that have been previously attributed to TET activity are not observed among Kawasaki patients.

Another manifestation that viral infection deregulates pathways, in which TET is involved, is related to the onset of diabetes among patients [103]. This condition has also been associated with changes in TETs 1-3 transcript levels and particularly of TET3 [104].

## 4. Discussion

In summary, we have presented the evidence that a large number of biological processes that have been associated with the TET activity and specificity are disturbed by SARS-CoV-2 (Table 1B). These involved the cell type- and organ-specific, neuronal, cardiac, lung, hepatic, and intestinal function. We propose that epidemiological characteristics of the infection, such as sex and age specificity, could be attributed to the critical role of TET activity in X chromosome inactivation and its developmentally dependent activity variations. Finally, we present evidence on the complex dependence of SARS-CoV-2 on hypoxia that could account for the discreet stages of the disease: Early-stage with limited symptoms and pronounced hypoxia and late-stage with serious complications. In addition, we present evidence that the existing age-dependent differences of TET activity might play a critical role in viral RNA deactivation among children, but the deregulation of this mechanism in adults might lead to modifications of the cell membrane environment and susceptibility of macrophages to SARS-CoV-2 attack (summarized in Table 1B).

**Figure 1:**
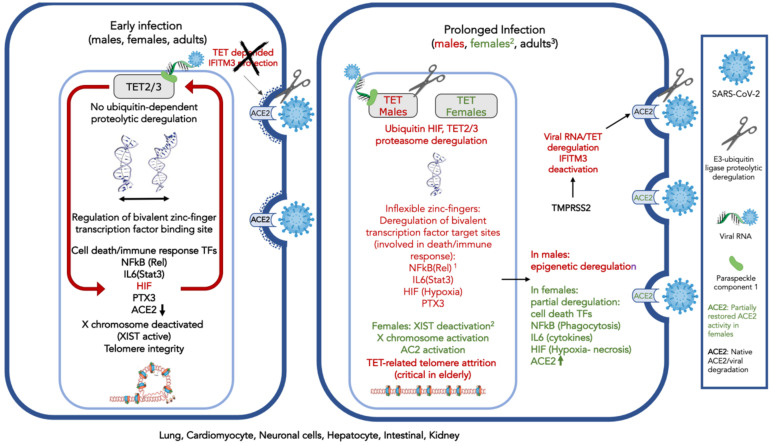
Conceptual model of severe acute respiratory syndrome coronavirus 2 (SARS-CoV-2) interference on ten-eleven translocase (TET) activity that is responsible for demethylation of bivalent promoter sequences, recognized by bivalent zinc finger-possessing transcription factors and G-quadruplexes in the lung, cardiomyocyte, neuronal cells, hepatocytes, intestinal, and kidney cells. Deregulation of TETs 2–3, is ubiquitin-proteasome dependent and might involve TET2 degradation. TET2 is recruited to the DNA by the viral RNA and a carrier (possibly Paraspeckle component 1, PSPC1). The protective role of cholesterol-transmembrane protein 3 (IFITM3) is dependent on TET2 activity and subject to the TMPR22, regulator of E3 protease activity, possibly similarly to the ACE2. On the left, early in SARS-CoV-2 infection: ACE2 expression decreases; HIF is expressed and deregulates TETs 2–3 activity by monoubiquitinylation and possibly degradation. IFITM3 is deactivated. Expression of cell-death and immune system-associated bivalent transcription factors regulated by TET are deregulated by the virus: NFkB and IL6 (through Rel and Stat3 demethylation) and other death-associated transcription factors. TET-associated neuronal functions can be affected; PTX3, which plays a role in the regulation of innate resistance to pathogens, inflammatory reactions, possibly clearance of self-components, and female fertility, is also subject to viral interference. On the right, after prolonged virus exposure: Acute inflammation and impaired immune response are observed; telomeres are affected. The X chromosome is activated via *XIST* gene deactivation; widespread perturbation of genome functionality is observed. 1. In dark red: Affected proteins, due to decrease, or modification of expression (e.g., splicing). 2. Only in females (in green): The X chromosome can be activated by TET-related *XIST* deregulation, leading to activation of immune response-related genes. Activation of the X chromosome would also involve ACE2 expression increase and partial protection from viral interference. 3. In children, contrary to adults, TETs 1–3 with complementary activities expressed during development, might compensate for viral interference.

Given the above-presented evidence with regards to TET involvement in viral response (Table 1A) and the evidence concerning the proposed association of TET biological activities and SARS-Cov-2 symptoms (Table 1B), a conceptual model for the SARS-CoV-2 interference is shown in Figure 1. TET 1-3 activity is known to play a major role in the lung, cardiomyocyte, neuronal cell, hepatocyte, intestinal, and kidney cell. TETs are subject to a ubiquitin-related E3 ligase-dependent proteolytic process involving the CUL4 ubiquitin ligase regulator(s) RBX1, and possibly factors DDB1 or DCAF1, also known as VprBP.

The SARS-CoV-2 viral RNA presentation to TET2 through a binding component (possibly PSCP-1) strongly activates HIF, leading to pronounced early hypoxia. The ACE2 susceptibility also increases, due to TET2-dependent IFITM3 deregulation, as well as proteolysis of viral spike protein S by TMPRSS2 (possibly also TET mediated). The proteasome activity and monoubiquitination probably mediate the TET imbalance.

TETs affect the flexibility of the zinc-finger binding sites present in bivalent receptors [34], several of which are related to cell death, others to homeobox protein binding, the immune response system regulation, hypoxia, and finally, with the X chromosome inactivation through the *XIST* gene expression that codes for the homonymous non-translated RNA silencer of the X chromosome. Moreover, TET activity is responsible for the nucleosome integrity of several genes, the regulation of G-quadruplexes and telomeres and is possibly related to the fidelity of the splicing process.

Viral infection could modify the critical TET activity, leading to deregulation of both expression and alternative splicing of several transcription factors, and protein isoforms are eventually also affected by the TET deregulation [34]. Only those which could be associated with known SARS-CoV-2 activity and symptoms are shown in Figure 1. ACE2 is affected, and viral presence increases.

After prolonged infection, TET imbalance introduces widespread perturbation of the functional state of the genome. However, *XIST*, which is responsible for X chromosome inactivation, might also be deregulated by this process, and X chromosome reactivation could be expected in females. X chromosome reactivation could lead to the expression of other X chromosome-related immune response genes, including ACE2. This process could partly compensate for the viral intervention, contribute to partial recovery of cellular functions, and provide “protection” of the female patients from the virus, compared to males.

TET also contributes to telomere stability, and its absence could enhance the compromised lymphocyte telomere integrity observed among the elderly [106]. Actually, the loss of telomere integrity, due to TET inactivation and concomitant PTX3 deregulation could be associated with reduced innate resistance to pathogens and could be common in other virus-associated pathological conditions [34].

The expression of the TET enzymes is developmentally regulated and attenuated by age. It is, thus, possible that among children, the TET expression and activity balance compensates for the viral activity.

These data introduce DNA demethylation as an additional parameter probably involved in deadly infections of SARS-CoV-2 and other viruses. It is expected that the proposed mechanism, which can account for many of the known viral symptoms, and can facilitate the design of new, possibly early, diagnostics and interventions, identifying intervention targets, predisposing factors, and possibly, developing improved prevention strategies.

## 5. Conclusions

Extensive research on TET activity provides multiple pieces of evidence that one of the tentative targets of SARS-CoV-2 is TET activity, an epigenetic regulator of the human genome, responsible for DNA demethylation in sequences regulated by bivalent zinc-finger transcription factors. According to the above, the consequences of SARS-CoV-2 infection might be long-lasting and possibly lead to irreversible modification of the genome among patients with prolonged recovery.

## Figures and Tables

**Table 1:** (**A**). Viral interference and TET activities. (**B**). Biological functions regulated by TET activity, vs. SARS-CoV-2 related symptoms and epidemiological characteristics of coronavirus disease (COVID-19).

A	
TET-Associated Viral Interference	Virus and/or Resulting Pathological Condition
1. Loss of genomic 5hmC, indicating TET2 downregulation [9]	Progression of adult T-cell leukemia/lymphoma (ATLL) associated with Human T-lymphotropic virus type 1
2. TET2 function as resistance factor against DNA methylation [10]	Epstein-Barr Viral infection
3. HIV Vpr protein mediates TET2 degradation through cellular CUL4A-DDB1 E3 ligase complex; IFITM3 deregulation by Vpr/TET2 [11]	Enhanced HIV-1 Env Processing and Virion Infectivity in Macrophages
4. TET is targeted for proteasomal degradation [12]	Hypoxia-related transcription factor in von-Hippel Lindau tumor suppressor (pVHL)
5. Viral RNA involvement in TET2 binding to DNA, in association with PSCP1 (Paraspeckle component 1) [13]	Endogenous retrovirus (ERV) control in pluripotent stem cells
6. Fumarate and succinate regulation of TET enzymes [14]	HIF target genes inhibited by TET via metabolic regulation
7. TET1 and TET3 expression [31]	Mice hypothalamus attenuation by age and activation by exercise
8. TET1 involvement in hypoxia-regulated processes [32]	Epithelial-mesenchymal transition
**B**	
**TET 1-3 Activities and Biological Functions** **in Different Tissues**	**COVID-19 Epidemiological Characteristics and Common Symptoms**
1. Brain differentiation [53], Neuronal degeneration [72], Global changes in spinal cord injury [75] TET-coordination of expression profiles in brain [81]	Ataxia [73], olfactory loss [61,62,63,76,77]; Delayed neurological recovery [7]
2. Hepatocyte differentiation, regulation [79]	Liver injuries [78]
3. Cardiomyocyte differentiation [83]	Cardiovascular complications [64]
4. Intestinal stem cell regulation [82]	Intestinal functional irregularities [78]
5. Pulmonary function, pulmonary arterial hypertension [70]	Pulmonary dysfunction
6. Age-dependent mC activity deviations [48] Age-dependent 5hmC reduction [105]	Age-dependent viral susceptibility [66] Limited symptoms of SARS-CoV-2 infection among children [67]
7. Telomere maintenance [92,93] aging telomere elongation [58], coiling [30,105] Tentative telomere regulation through inflammation control by TET [34]	Telomeric attrition in elderly patients in intensive care units [94], Telomeric compromise in aging lymphocytes in COVID-19 patients [95] Telomeric length and COVID-19-dependent lethality [106]
8. X chromosome inactivation indirectly regulated by TET through *XIST* gene expression (responsible for X inactivation) [57]	Lower mortality rates among women [65]
9. Regulation of X-chromosome linked cytokine expression through PTX3 regulation (tentatively under TET regulation [34]	Cytokine overexpression [21,100,101]
10. TET regulation of metabolically regulated DNA epigenetic processes (cardiomyocyte) [85] and activation of the citric cycle [84,91]	Pronounced hypoxia [22]
11. ΤΕΤ association with succinate, fumarate and metabolic control [14,87]	Obesity and diabetes risk factors [8]
